# Towards a more comprehensive approach for a total economic assessment of vaccines? 1. The building blocks for a health economic assessment of vaccination

**DOI:** 10.1080/20016689.2017.1335162

**Published:** 2017-08-31

**Authors:** Baudouin Standaert, Rino Rappuoli

**Affiliations:** ^a^ Health Economics, GSK, Wavre, Belgium​​​​; ^b^ Research & Development, GSK, Siena, Italy

**Keywords:** Budget, economic evaluation, incremental cost-effectiveness ratio, societal perspective, vaccines, value assessment

## Abstract

​Since we were born, we all take preventative actions to avoid unpredictable adverse conditions. Some actions are done automatically. Others require a conscious choice , either for personal or social benefit. A distinction can therefore be drawn between non-active and active prevention, and between individual and social prevention. Active prevention requires making a choice in time, effort, and cost. We call it an economic choice. Vaccines belong to the group of active and social prevention. Because a vaccination program is an economic social choice, how should it be valued, and what cost should we pay for? To date, the economic evaluations developed for treatment have been applied to vaccines. However, over 25 different characteristics differentiate vaccines from treatment. For example, the benefit of vaccination is measured at the population level not at the individual level, the main effect of prevention is societal and not an individual-based gain only, and the biggest hurdle to implement a new vaccine is the initial budget investment and not so much its estimated ‘value for money’. This makes the current application of incremental cost-utility analysis difficult for vaccines for a comprehensive evaluation. New approaches may be needed to capture the full economic benefit of vaccines.​

## Introduction

Health-economic evaluation of newly introduced healthcare interventions is now widespread in healthcare decision-making processes across the world [1]. It is part of the Health Technology Assessment (HTA) programmes, which include issues such as clinical effectiveness, safety and cost-effectiveness within a broad field of social, ethical and legal aspects required for a new intervention to obtain local approval of reimbursement [2]. Within that framework, the economic method currently most used for the evaluation of vaccines is the incremental cost–utility analysis (ICUA), which was developed for the evaluation of new therapeutic interventions such as drug treatments [3–7]. Although the method is theoretically transferable from treatments to vaccine prevention, there are issues which mean that it may be incomplete or too narrowly focussed on one benefit mainly [8]. As a consequence, ICUA may undervalue the importance of prevention for healthcare and for society. For example, traditional ICUA focuses on health benefit at the level of the individual patient receiving treatment, expressed in quality-adjusted survival gain and/or in symptom relief. In contrast, vaccines are implemented across a whole population at risk, with wider benefits that may not be adequately captured in the conventional health-economic evaluation methods of today [9].

That there are issues with the evaluation methods proposed for vaccines is indicated by the recent initiatives taken by international groups such as the International Society for Pharmacoeconomics and Outcomes Research (ISPOR) or HTA international (HTAi) to install working groups or task forces to evaluate in greater detail the economic assessment of vaccines opening the view from cost-effectiveness evaluation, to optimisation modelling and fiscal modelling.

This first of three papers describes the key methods currently used in the health-economic assessment of new medical interventions when introduced, with a focus on vaccines. It starts by defining prevention with the role of vaccines as an active preventative intervention and considers the difference between vaccines and vaccination programmes. It highlights the differences between vaccine prevention and therapeutic drugs. It then moves to health economics, with the definition of economic value as used in healthcare, and the methods developed for collecting such information. It briefly touches the ways economic assessment in healthcare has evolved over time. It finally discusses the optimal positioning of the two worlds of prevention and treatment in the current armament of healthcare interventions.

## What is prevention?

**Figure 1.**
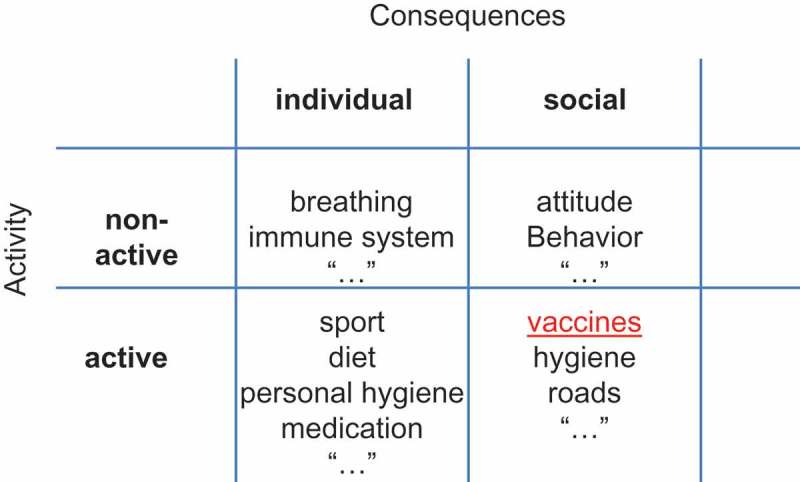
Classification of prevention.

Dictionary definitions of prevention include ‘the act or practice of stopping something bad from happening’ [10]. A more comprehensive definition would precise that prevention is a risk-mitigation mechanism to reduce at best bad conditions that might happen with a certain probability but about which we remain uncertain when precisely it might occur. Prevention activities can be categorised into active or non-active, and prevention consequences can be categorised as individual or social (see Figure 1).

Non-active prevention is ubiquitous. People generally try to live with as little harm as possible. The human body is full of sensors that continuously detect potential threats and take automatic preventative actions without hesitation, such as the reflex of immediately retracting the hand on contact with a hot plate. The immune system is a sophisticated preventative programme that continually protects against harmful external pathogens. Active prevention differs from non-active in that there is always an associated extra-effort to be made. It is therefore an economic choice in time to be assigned to do it and in payment required. The cost of undertaking active prevention varies from cheap to expensive, and the consequences of *not* undertaking it can also range from minor to major. Preventative actions can take many different forms, can operate on timescales from immediate to distant future, and can range geographically from single individuals to worldwide effects. Most disease preventions belong to the domain of active prevention. It includes primary prevention (before signs and symptoms appear), secondary prevention (early on in the disease process), and/or tertiary prevention (to avoid aggravation) [11].

In this paper we also added the dimension of individual and social consequences (Figure 1). This distinction is important as it helps to define whether the effect of a preventative act should be evaluated individually or by group.

## What is a vaccine and what is vaccination?

Vaccines have been developed mainly for the prevention of transmissible infectious diseases. They can act in two ways. One is by avoiding infection taking place, the other is by avoiding reactivation of an infection such as may happen in the case of zoster infection. Two age groups are particularly vulnerable to infectious diseases: young children whose immune system is not yet fully developed, and elderly people because their immune system tends to weaken with age [12,13]. Vaccination consists of the administration of controlled antigens, small particles that are foreign to the human body and that mimic in an attenuated form the wild-type antigens found on specific infectious microorganisms such as bacteria and viruses. The vaccine antigens stimulate the body to mount an immune response, creating antibodies and activating immune cells that can swiftly destroy infectious organisms before the infection or disease becomes established [14]. Today, about 30 diseases worldwide are vaccine-preventable [15].

Developing a vaccine with good clinical efficacy results in randomised clinical trials is only the first stage. A thorough vaccination programme must be implemented, and will be considered successful when high vaccine coverage of the at-risk population is maintained over time. Only then will the full benefits of the vaccine and its vaccination programme become apparent.

Vaccination is a unique form of healthcare intervention that is most often developed and promoted at country level by public health institutions [16]. Unlike therapeutic interventions, which aim to cure or alleviate existing disease symptoms in an individual patient, the focus of vaccination is on prevention at the population level, making it part of the public health domain [17]. Vaccination requires medical providers to search actively for unvaccinated individuals to administer the vaccine. Passively waiting for people to present for vaccination would be inappropriate for a successful vaccination programme [18].

Many different implementation scenarios exist, depending on the market and the process of acquiring new vaccines at country level. A few broad patterns can be observed (Table 1), although mixed scenarios occur. At one extreme is the private market where the payer is private, the vaccine administration may happen in a private environment, and the initiative is private.

**Table 1.** Vaccination extends beyond vaccine availability.

Activities	Process handling options					
Registration	Central (FDA-EMA) /local	List cost/market cost/reference cost	Clinical dossier (plus economic)	+ Local data	Access to pharmacy or to GPs	Full value/ICUA
Acquisition	Tender	Reimbursement	Co-payment	Out-of-pocket	Mixed	Private
Stockpiling	Warehouse	Wholesaler	Pharmacist	Local vaccine clinics	GP level	
Transport	Cold-chain requirement	Bulk transport (by ship, aeroplane, trucks)	Local transport (by cars, motorbikes, horses, on foot)	New techniques, e.g., drones		
Administration	Private clinics	Public vaccination centres	Organised school programmes			
Waste management	Organised through pharmacies					

EMA, European Medicine Agency; FDA, Food and Drug Administration; GP, general practitioner, ICUA, incremental cost–utility analysis.

The only governmental intervention here is that the product is registered and authorised to be sold in the country at an approved list price. Rotavirus vaccination in the Netherlands is an example of this situation today [19]. The vaccine can be bought through a private pharmacy, although it might take some time to obtain it because it is not immediately available. There is no recommendation issued by the health authorities, no reimbursement, and no promotion is permitted [20]. The other extreme is that everything is regulated by the health authorities, from acquiring the vaccines through tenders, distribution, local organisation of specific vaccination sessions for children at different age points, to post-vaccination follow-up. Everything is paid for by the authorities and the only request to the consumer is to be there at the moment of the planned vaccine administration. The rotavirus vaccine situation in the UK is a perfect example of that condition now. Between these extremes there are many possible variations, such as reimbursement instead of tender with and without co-payment, normal physician visits to the general practitioner (GP) instead of organised visits or direct access in pharmacies, specific sessions, and prevention cards for monitoring the full vaccination schedule per person instead of no follow-up. Each of these variations has pros and cons [21]. In general, if a vaccine implementation programme is already well established, this creates many advantages for successful implementation of an additional new one, because the existing system can be utilised, adjusted where necessary, can better estimate the cost and time required, and can handle follow-up of the new programme. If no established programme is in place and the authorities plan to organise a tender for the first time to obtain a cheaper vaccine price, a programme of distribution and administration will need to be organised. It is likely that under such circumstances considerable waste may occur and the efficiency of the whole programme may suffer [22]. The same can be said about mixed programmes, in which the vaccine is fully reimbursed but no administration is organised except that GPs are able to do it. This may result in vaccines being unavailable at the GP level, requiring additional consultations and visits to the pharmacy, which in turn may inhibit implementation of a new vaccine and limit the coverage rate.

Problems of implementation are even more acute in the developing world, where some regions may be difficult to reach and a cold-chain process must be put in place, which means that the vaccination process may be more expensive than the vaccine itself [22]. New transport techniques such as drones may have the potential to become game-changers in such environments, but at present this is not so well established except in the example of Rwanda at the moment [23]. Implementation is a key aspect of successful vaccination, and may imply additional costs if good logistics are not already in place.

As an example of the influence of implementation on vaccine coverage rate, the human papillomavirus (HPV) vaccination status of young girls in the UK can be compared with the USA. In the UK, HPV vaccination was well organised through school medical programmes and the initial coverage rate was 95% or more [24]. In the USA, the vaccine is available in pharmacies, but the initiative for vaccination had to be taken by the parents and the individual GP, and there the coverage rate is up to 35% among the same target group [25].

## Prevention versus treatment?

Prevention is the overwhelming norm of our day-to-day activity behaviour in our life. We apply prevention for many different reasons that are not always directly or immediately linked to gaining better health. Our norm-setting in prevention is therefore large and open, from avoiding what doesn’t seem good, to being defensive and not acting, or gaining, to success. In contrast, treatment is the exception when prevention fails under certain conditions. However, treatment has a norm-setting that is very focussed and restrictive. It restores at best what was not going well. In medicine the primary norm-setting has evolved over time, with treatment now often being the reference of the medical act while active prevention became the exception. Because of that we evaluate prevention today as a special case of treatment while it normally should be the other way around, but reversing that perspective is difficult to achieve [11].

Active prevention has reached some of the most successful interventions of medicine in history, particularly in public health [26]. Quarantine or effective isolation measures limited the spread of some specific diseases even when the real root cause or the mechanism of spread were not well known, like the cholera epidemics in London in the 1850s [27]. Such measures were applied against communicable or infectious diseases, which at the time were the main cause of preventable early death [28]. As understanding of infection developed, preventative actions became more specific and more effective, and attempts were made to make interventions more efficient by considering specific risk groups, administrative routes or new techniques such as immune stimulation [29].

Prevention was thus very efficient with numerous overwhelming successes [30], and one might therefore expect that further enhancement of prevention would be the preferred method in developing medicine. Indeed, once it was recognised that the main cause for infectious diseases was in the microbiology, public health institutes were set up, where bacteria and other parasites were identified and isolated to understand their growth and spread using epidemiological methods of investigation [29]. However, people suffering from acute conditions still needed immediate help that could not be provided by prevention but only by the delivery of direct medical care. For the individual, waiting until symptoms appear and then being treated by a doctor, after which the symptoms disappear, is a course of events in which the relationship between intervention and benefit is obvious. Everyone involved in this relationship receives an immediate direct benefit, provided that the services delivered are affordable and successful. The patient gains the satisfaction of having the symptoms resolved, and the doctor gains the satisfaction of a successfully treated case. In contrast, prevention often does not show a short-term direct benefit. When treatment and prevention are in competition for resources, this difference in direct measurable benefit may be an important issue. Treatment and cure became popular with the appearance of effective drugs on the market and easy access for those most in need, as illustrated by the use of antibiotics against infections [31]. Looking at the money spent on treatment versus prevention today, it is apparent that treatment won the competition by a large margin [11]. In the developed world a whole medical corps is ready to act day and night when a patient asks for help to alleviate his/her symptoms. There is a full medical infrastructure available and the programme is easily accessible, with payment and costs covered for many people. In addition, society and politicians have also pushed the healthcare programme to move into the curative direction, via a system of social security development to make cure and treatment affordable for many in the population.

## Differences between vaccines and therapeutic drugs

A list of ways in which vaccination differs from therapeutic drugs is presented in the Supplementary File 1 and summarised in Table 2. The list is not exhaustive, but helps to illustrate why vaccines may need a different approach to economic assessment. Each element in the table may have an opposite view for vaccine prevention and for drug therapy. For example, the objective of vaccines is prevention, while for treatment it is cure, care or symptom relief, very different goals.

**Table 2.** Summary of the differences between vaccines and drug treatment.

Product (9)	People/healthcare (11)	Finance/budget (8)
Objective	Target group	Financial
Safety	Environment	Budget
Efficacy	Impact	Purchasing
Outcome measures	Access	Investment
Reactivity	Societal	Market type
Duration	Organisation	Third-party payer
Switch	Evaluation	Risk-sharing programmes
Focus	Monitoring	Decision-making
Production	Acceptance	
	At-risk	
	Information	

As mentioned earlier, successful treatment is associated with an immediate, clear and direct benefit, whereas the benefits of prevention may not be apparent in the short term. A therapeutic intervention is normally administered after disease symptoms have appeared with sufficient severity to prompt the patient to seek treatment [32]. Success is readily recognisable by the patient and/or the doctor, who should see cure or improvement over a short time frame. The effect of the intervention is limited to a narrow social group, mainly the affected individual, and to the later stages of the infectious disease process, after the patient has experienced symptoms for a period. Treatment evaluation also often stops when medical attention is no longer needed, thus missing any post-treatment period of residual impairment before full recovery. Furthermore, medically administered therapeutic interventions do not affect mild disease episodes for which medical attention is not sought [33]. Thus, medical treatment addresses only part of the total disease burden, although as it is the part most immediately obvious to the patient, it is the simplest part to recognise and to measure.

In contrast, vaccination targets a larger fraction of the disease burden than medical therapeutic intervention, as the vaccinated individual can be protected against mild disease episodes in addition and against the whole of a disease episode including the period before receiving treatment and residual post-treatment impairment. The accumulated quality of life (QoL) gain with a vaccine should therefore be higher than for a therapeutic intervention with similar efficacy. However, the benefit is less easy to recognise and measure, because the benefit of vaccination is that something harmful – an infectious episode and its consequences – does *not* happen. The experience of prevention as a measurable benefit reported by an individual is not possible to capture: he cannot precisely identify when the prevention moment occurred and whether there was a change in his QoL. He often remains indifferent to the process because of not knowing that prevention happened. And that is a big issue that the benefit of prevention can only be measured in comparison with data outside an individual experience of the new intervention.

Considering the differences outlined above between vaccine prevention and drug therapy, it is not so difficult to recognise that the economic value assessment may differ between those two worlds, even though both programmes may have the same ambition of improving the health condition of a population. Conventional assessment comparing the two interventions can be conducted to the extent of incremental analysis of the benefit generated by each intervention over the other. However, the economic assessment of vaccines is more subtle than for drugs for four broad reasons:

- most vaccine benefit is observed at the *population* level, because it includes externalities such as herd protection and the reduction in antimicrobial resistance (AMR) which are additional benefits for people other than the at-risk group targeted for vaccination (i.e., caregivers, employers, payers), and the overall economic and welfare improvement resulting from a permanently healthier population who may therefore be more active and productive;
- the *societal* consequences of vaccination are bigger than with any treatment, because vaccines avoid many disease events that normally do not receive medical attention but may cause important productivity losses for non-professional caregivers. This occurs especially for disease episodes among children (parents) and the elderly (family members). The vaccination impact can also be substantial at the level of overall disease management in the healthcare sector; for example, improvement in hospital quality of care after the introduction of the rotavirus vaccine [34]. A vaccine introduction with an immediate high coverage rate and an instantaneous impact normally introduces an imbalance in healthcare delivery, because resources have to be switched from care to prevention and that has consequences for the existing care delivery [34];
- the *initial investment* for introducing a new vaccine may be large for the payer or decision-maker, without much certainty about the short-term benefit. Thus, implementing vaccination is a more risky decision that often needs to be defended at a higher level than the health ministry in a country. This higher decision level may use economic evaluation techniques and vocabulary that differ from those developed for health economics. Therefore, one needs to be ready to provide the required data/analysis and results that other decision-makers, such as the Ministry of Finance or Ministry of Planning, may be more familiar with or more willing to accept;
- *timing* in performing the economic assessment of vaccination will give a different result if the objective is disease control (short-term effect), or elimination and eradication (long-term effect). This shift in overall objective through the implementation of one type of intervention such as vaccination is not seen for any other therapeutic goal.

## The role of the health technology assessment

Decision-makers in healthcare who are the main payers of the system are today exposed to some critical issues [35]. They face budget limits despite the higher demand for more delivery and at the same time they may see the supply of more new and more costly interventions seeking access to the healthcare market asking for reimbursement at a high price. Those two different financial forces (less budget for more demand and more supply at a higher price) oblige the decision-maker to channel each request for new investment through an evaluation process, assessing the critical questions whether the new asset will work in the environment proposed (effectiveness in real-life situations as opposed to efficacy from randomised clinical trials); and whether it is worth buying the new asset (cost–efficiency evaluation) given the limitations that exist.

**Figure 2.**
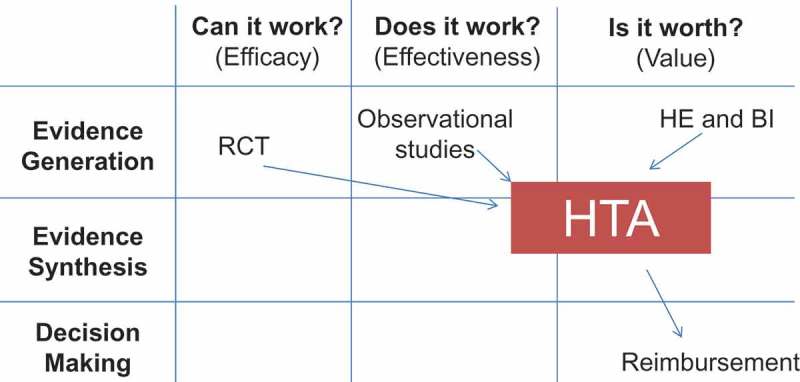
Positioning an HTA programme within healthcare. BI, budget impact; HE, health economics; HTA, health technology assessment; RCT, randomised controlled trial. Adapted from Luce et al., 2010 [36].

Most healthcare organisations have now implemented HTA programmes that help to answer both questions. Luce et al. have defined where HTA should be positioned using a matrix approach of questions to be answered and decisions to be taken (Figure 2) [36]. It is critical to see that HTA is more than focussing on cost–effectiveness analysis. It also tries to consider what may happen with the new asset in real-life conditions, and whether the money spent is good value for health and for the healthcare programme.

HTA programmes have now been endorsed by many countries, especially when the healthcare programme is sponsored by public funding as in most European countries. Special bodies have been installed to evaluate the HTA programme in order to help the decision-maker, e.g., the National Institute for Health and Care Excellence (NICE) in the UK, the Institute for Quality and Efficiency in Health Care (IQWIG) in Germany, the Canadian Agency for Drugs and Technology in Health (CADTH) in Canada, among others. Variation exists between HTA programmes in different countries, although harmonisation is sought through organisations such as the International Network of Agencies on Health Technology Assessment (INAHTA). Through the country-specific bodies, value measurements of new assets are evaluated and reported among members, allowing comparison of results across countries.

Some countries have installed specific organisations to assess new vaccines, e.g., the Joint Committee on Vaccination and Immunisation (JVCI) in the UK or the Standing Committee on Vaccination (STIKO) in Germany. The WHO is proposing to develop National Immunisation Technical Advisory Groups (NITAG) in each country and provides support through the regional Supporting National Independent Immunization and Vaccine Advisory Committees (SIVAC) initiative [37]. The programme of a NITAG does not officially include HTA assessment, but all the elements of HTA are part of the NITAG evaluation process. An example of a NITAG programme in the Netherlands is presented in Table 3 [38].

**Table 3.** Programme of the National Immunisation Technical Advisory Group (NITAG) as developed in the Netherlands.

	
The role of NITAG	Preventing public risk
	Creating equal access
The function of NITAG	
Customer	Defining: • disease burden • therapeutic options • higher risk for fatality • risk groups who cannot protect themselves
Product	Defining: • effectiveness • real-life protection gained from vaccination • adverse effects assessment • acceptability • efficiency (cost, technical, social) • urgency

## Defining value

Having considered the organisation, the timing and the questions to be answered when introducing a new asset in a country, the next step is to understand what the evaluation process will be. The value of the new asset compared with the existing situation is a critical element. In a normal economic evaluation of a new consumable good coming into the market, the concept, definition and quantification of its value is simple and precise. If the payer is the beneficiary or the consumer, the economic value will include three characteristics: the *intrinsic* (= cost of goods), the *instrumental* (= what it is used for), and the *inherent* (= brand-specific) value (Table 4) [39]. All three can be quantified and expressed in one evaluation unit: money. The value helps to define the price of a new product at launch if the market is functioning well, i.e., if full information about the product is available for everyone, fair competition is present, and easy and uniform access to the product by the consumer is guaranteed. Under such circumstances the market value of a consumable good is explained by its *instrumental* plus its *inherent* value, defined as perceived by an external observer, the consumer who pays [40]. The price therefore perfectly reflects the product value.

**Table 4.** Value assessment in economic evaluations.

**Open Market** Cost=Value Payer = Consumer		**Non-market** Cost≠Value Payer ≠ Consumer						
**Demand/Supply**		**Cost-Benefit Analysis**		**Cost-Effectiveness Analysis**				
		*Investment cost*	*Willingness to pay*		*Preference*			
			Direct	Indirect	Unscored			Pre-scored
					Certainty (value)		Uncertainty (utility)	Multi-attribute health status (questionnaire)
Intrinsic	Cost of goods	Human capital approach	Revealed Consumer behaviour Travel cost method Hedonic pricing method	Stated Survey Contingent valuation method Discrete choice experiment	Scaling	Rating scale Category scaling Visual analogue scale Ratio scaling		Quality of well-being EQ-5D Short Form 6D Health utility index
Instrumental	What it is used for							
Inherent	Brand-specific				Choice	Time trade-off Paired comparison Equivalence Person trade-off	Standard gamble	
	

EQ-5D, EuroQol of life five dimensions questionnaire.

The situation becomes more complicated when the price no longer plays that role, because markets are not functioning well or do not exist: the non-market situation. This is the case with healthcare, where easy access is not always guaranteed, product knowledge is asymmetric among those interested (the payer, the seller, and the consumer), there is much uncertainty about the benefit of a new intervention, and most importantly the consumer is often not the payer and the payer may impose rules on price-setting for reasons that are not market-driven, such as reasons of equity access. Thus, the value assessment has to differ from that seen in an open market.

Currently, we like to operate under the terms of inherent value assessment in healthcare by promoting ‘value-based’ pricing [41]. This requires defining what value means and how to measure it in non-market conditions, where the price paid for a medical good does not reflect its value to the consumer.

In a non-market situation, the value of a new intervention can be expressed in non-monetary units on the condition that the same impact measure can be compared across different medical goods available. Apples should be compared with apples, not with pears, and that can sometimes be a challenge. It would have been easier for decision-makers if value were expressed in monetary terms. However, there was reluctance to do so when the development of health-economic evaluation tools began some 40 years ago, as the major benefit of health interventions was/is health gain and it was difficult for the medical corps to accept expression of this in dollar units [42].

Another problem that arose at the start of the development of economic evaluation of new medical goods was the method selected to perform the economic assessment. As healthcare operated in a non-market environment with a social dimension to get maximum equity of access, the one approach to economic assessment of public projects that was well-developed at the time was the cost–benefit analysis (CBA) [39,43]. This was used in investigating major non-market projects such as water resource projects or transportation projects, the benefit achieved outweighing the investment cost. However, in CBA the benefit must be expressed in money terms. One approach often used was the human capital method, measuring the new healthy time obtained by the new intervention presented as a work production improvement expressed in money. The other method was the willingness to pay (WTP) assessment, which attempts to measure how much consumers want to pay for the extra benefit they can get from obtaining clean air or water. Different methods have been developed to collect WTP information. They are classified into direct and indirect methods and have been used extensively in the assessment of environmental problems that tackled the same issues as healthcare of life-years and quality-adjusted life-years (QALYs) gained.

In that respect, much more research has been undertaken to develop the right instruments to collect the information in the environmental domain than has been done in healthcare. There is much to be learned from CBA conducted in environmental contexts that could be applied to healthcare programmes today [44].

The proposed alternative to CBA, avoiding the expression of benefit in monetary terms, was incremental cost–effectiveness analysis (ICEA) and ICUA, which will be discussed in the next section. The latter is the one most widely applied in healthcare today [45]. In this context the major issue in value assessment was around the benefit measure to be selected. Much debate centred on the technique used to quantify the QoL linked to the health gain obtained, whether a value with precise criteria should be measured or a utility as defined by Morgenstern and von Neumann [46] which includes risk assessment with uncertainty levels. The results obtained will differ depending on concepts and methods used. As with CBA, the approaches can be categorised depending on whether a score needs to be initiated or if the score already exists. The selection is often influenced by budget restriction to obtain the information, and by the motivation of the researcher who may be convinced that utility should be measured instead of a value. Table 4 summarises value assessments for open-market and non-market conditions. The key point is that in ICUA the value concept in an analysis depends on the method used, and is mainly driven by the quality level of the health gain obtained.

The value assessment of vaccines using ICUA has been most often performed using an approach similar to evaluation of treatments, expressing value as QALY gained or disability-adjusted life-year (DALY) avoided [47].

## Challenges in defining the value of vaccines

Several problems make this value assessment approach potentially less strong and/or less complete for vaccines, resulting in large variations reported for the same vaccine in an equivalent environment.

- Vaccines produce prevention or avoidance of events. This is very difficult to measure or to be reported by a vaccinated person who will not experience any difference when he doesn’t get the event as he will not know that this is happening at a precise moment. The benefit is hidden and will most easily be reported if the comparison can be made with a separate group of individuals who are not vaccinated but exposed to the same infection risk.
- In addition, most vaccines have been introduced in infants and children. To investigate the patient harm that will be avoided by the vaccine, surrogates have to be interviewed, usually parents or healthcare professionals [48]. This may create broader value perception shifts than obtained among those who may directly benefit from vaccination.
- Third is the question of whether harm in those close to vaccinated individuals should also be collected and reported. The unit of value should not be the individual to be vaccinated, but the household that may function better if the disease burden is reduced through vaccination [49].
- Fourth, the benefit is often reported among those who have been vaccinated, while vaccines have the ability to create positive externalities among unvaccinated populations, expressed as herd protection effects and the reduction in AMR that may appear with excessive or inappropriate use of antibiotics [50].
- Fifth, vaccination fulfils multiple functions besides the dramatic initial reduction in disease burden. It is the best instrument to control the spread of infectious diseases over time that may lead to elimination and/or eradication [51]. This additional value potential has been poorly evaluated and not included in vaccine value assessment because eradication/elimination is rare and it is uncertain how to express this value [52].
- Sixth, if the disease burden is high and shows periodic peaks, it is likely that high initial uptake of a vaccine with externalities in the unvaccinated population may avoid many events during a short time period, which may create an imbalance in care delivery and a new externality of improvement in quality of care (QoC) [34]. This type of benefit has also been poorly reported in the value assessment of vaccines [53].
- Seventh, the general population may feel more secure if they know that they are better protected against a deadly infection, such as meningitis in children, by high vaccination coverage of the at-risk population in their direct environment [54].
- Eighth, reduction of absenteeism at work, which may result in a higher cost gain for society than the reduction in medical resource use, can be measured with vaccines and is often missed from reporting [55].
- It should be noted that mothers with fully vaccinated young children may benefit from better time management, an important benefit in the multi-task environment of this societal group [56].

**Figure 3.**
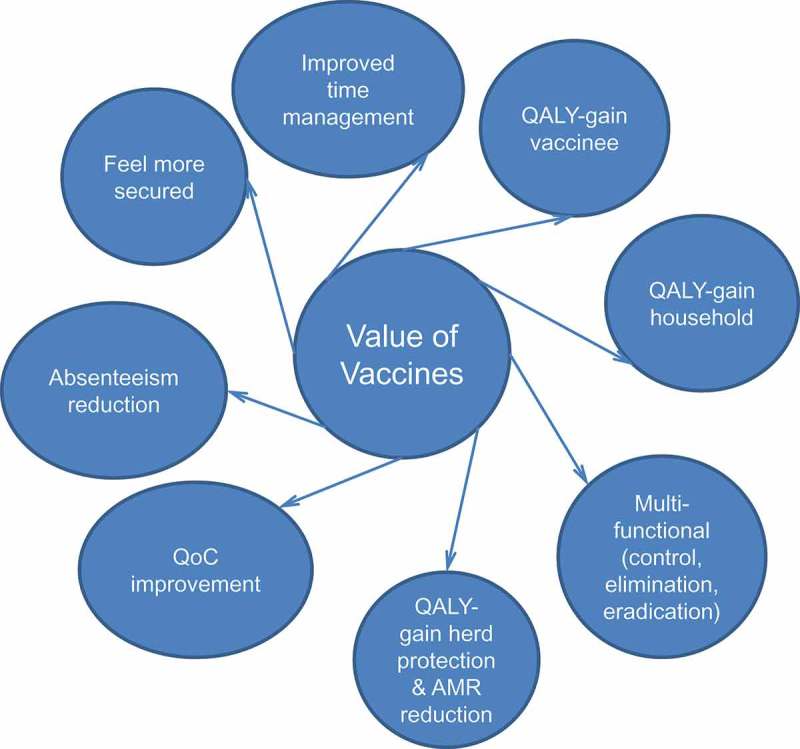
Additional value characteristics and gains with vaccines. QALY, quality-adjusted life-year; QoC, quality of care; AMR, antimicrobial resistance.

In summary, vaccines have a broader scope of value impact than just a gain in quality health at the individual level (Figure 3). They mainly operate at the population and societal level and therefore their value cannot be evaluated using a technique limited to clinical value only, such as QALYs gained [57].

Finally, the value perception of vaccines must often be split between the payer and the consumer. The forces driving value perception for consumer and payer are different, and therefore their priority settings will also differ. The payer normally looks first at the overall budget, considering the affordability of adding a new intervention that procures more total value for the population for whom he is financially responsible, or what must be displaced from the portfolio to add the new intervention. The beneficiary is not always sensitive to the direct monetary aspects of an intervention if payment has not been required, but wants easy access, no harm or side effects, and good protection [58]. Information relevant to the consumer value is often collected and applied to a payer model to calculate the incremental cost–utility. But is that the right way to deal with?

## Current economic assessment of new medical interventions

Historically, the economic evaluation of new interventions in healthcare primarily used ICEA or ICUA [42]. There was a need to identify ways of setting priorities to respond to budget constraints and to allocate resources more efficiently. The first rationale for developing economic guidance on pricing for new medical interventions was preferential selection of new products that could generate additional benefit compared with existing options. ‘Me-too’ or equivalent-value products were allowed, but they were often clustered at particular cost levels as they were used to create competitive cost comparisons to reduce the price [41]. Later developments looked at quantifying how much extra should be paid for the quality of the clinical benefit gained [59]. Payers seemed at first to be mainly concerned with finding ways to quantify reasonable extra payment, rather than searching for cost reductions with the new interventions. From initially ranking interventions by mean cost–effectiveness results – the lower the result, the better the ranking – and selecting the better-ranked interventions first, payers moved to incremental cost–utility ratio (ICUR), extra cost per QALY gained [60]. This new approach inherently accepts that increased health gain would be associated with extra payment (Table 5). Interventions were funded in order of ICUR until no budget remained [61]. However, this raised issues of equity, as costly interventions for rare diseases with high ICUR could rarely receive funding, which was inconsistent with the wish to make healthcare accessible to as many people as possible [62].

**Table 5.** Ranking different interventions in function of incremental QALY analysis or incremental cost–utility ratio for their selection with a threshold and/or a budget limitation (hypothetical case).

Intervention	Cost	QALYs	ICUA	Ranking	Incremental cost	Incremental QALYs	ICUR	Ranking	≤ threshold 500/QALY	Incremental budget	≤ Total budget 250,000
A	50,000	500	100	2							
B	70,000	900	78	1	20,000	400	50	1	X	70,000	X
C	150,000	1,050	143	3	100,000	550	182	2	X	220,000	X
D	300,000	950	316	4	250,000	450	556	4			
E	350,000	1,100	318	5	300,000	600	500	3	X	570,000	

ICUA, incremental cost–utility analysis; ICUR, incremental cost–utility ratio; QALY, quality-adjusted life-year.

**Figure 4.**
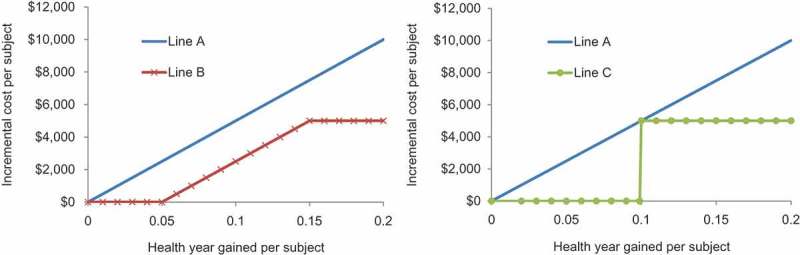
Incremental analysis (Line A) versus other threshold options (minimum health gain to be achieved – Line B, and individual level – Line C).

Later, thresholds were introduced, defining the ICUR considered acceptable by decision-makers [63]. Different ways to define the threshold were proposed, from using the last intervention accepted for the budget defined (supply-induced setting) to external assessments using WTP evaluation methods (demand-induced setting). The threshold approach could be more restrictive than the simple ranking system in assigning the available budget when the ranking is not able to do so, but it has the same weakness for high-ICUR products in rare diseases. However, a main reason for introducing a threshold is that a nice ranking of all the different interventions often doesn’t exist and therefore we cannot precisely define what is the last cost-effective intervention approved within the budget line. The threshold should help to define this indirectly [64]. In addition, it focuses the evaluation on more QALYs leading to extra payment (Line A in Figure 4). The threshold is the same at any point of the incremental benefit. However, other threshold options related to that benefit could have been considered: a minimum health gain to be specified or an absolute cost increase barrier to be applied, above which no extra payment is made for extra benefit achieved (Line B in Figure 4). At the extreme, a threshold value for health gain at the individual level could be introduced, which will be compensated by a fixed extra cost because of budget constraints (Line C in Figure 4).

When ICUR was introduced 45 years ago, no such alternative thinking was considered. Line A in Figure 4 still governs the analysis, paying more for more health gain. In relative terms, there is a maximum cost per healthy year gained and therefore expenses are restricted to below the threshold value line. This approach also implies that payers must fund any intervention whose ICUR is below the threshold, regardless of whether sufficient budget is available. If adequate budget is not available, this puts pressure on other interventions that are cost-effective but must be displaced to respect the budget limit if new interventions with more QALY gain, that meet the threshold, are introduced. A budget limit may be more appropriate in health-economic assessment of alternative interventions, or at least budget evaluations should be considered in addition to ICUA to obtain a more complete economic analysis [65].

**Figure 5.**
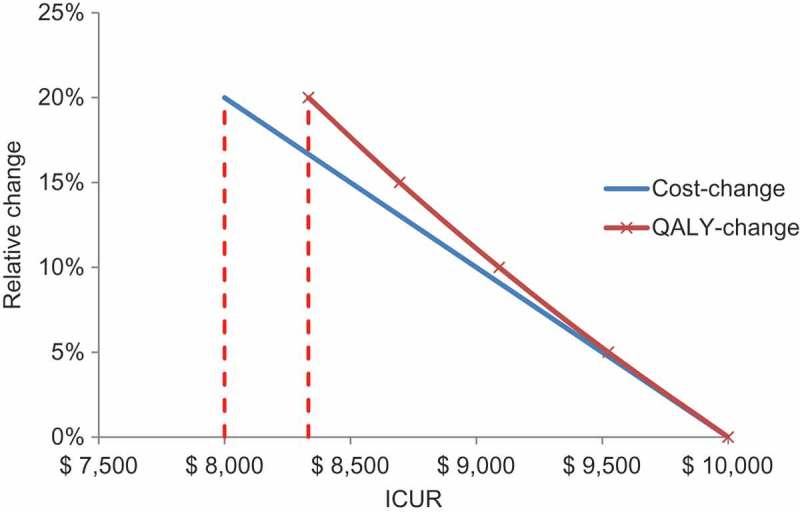
The relative change in cost- or quality-adjusted life-year difference on the incremental cost–utility ratio result. ICUR, incremental cost–utility ratio; QALY, quality-adjusted life-year.

ICUR with QALYs as the main outcome measure, often in conjunction with a cost-utility threshold or range, is now the established approach to the evaluation of new medical interventions, used by HTA authorities such as NICE for new treatments and the Joint Committee on Vaccination and Immunisation (JCVI) for new vaccines in the UK [66]. The academic health economists who initially developed the cost–utility analysis techniques did not conceive of differentiating between types of gain for healthcare and society, and therefore benefits other than QALYs gained were considered poorly or not at all [67]. All other potential benefits should be expressed as a QALY value or a cost offset, with a cost gain preferred over a QALY gain if a choice can be made. That is because cost differences are generally larger in absolute terms than QALY gains for the same relative value change (Figure 5; 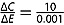). An additional consequence is that any societal benefit that cannot be expressed in those measures has tended to be disregarded or undervalued. This is not considered a major limitation when assessing and selecting therapeutic interventions at the level of an individual patient, which is the context in which ICUA was originally developed. However, it has limitations when applied to preventative public health initiatives such as vaccines, which differ from therapeutic interventions in important ways as discussed.

Finally, it is also important to highlight that with vaccination the ratio concept of the ICUR is stretched to its limits in two directions. The incremental QALY gain per person vaccinated may be very low in the developed world, which tends to increase the maximum achievable cost-effective result exponentially instead of linearly with cost. But when investment in healthcare is poor, as in developing countries, the cost-offset could be near zero, which dramatically widens the range in QALY gain and in the cost that is still cost-effective under the local threshold [68]. So, the meaning of ICUR results for vaccines should be evaluated very carefully. Introducing clear definitions about what to expect from vaccines could be very helpful.

## Discussion

This evaluation of the building blocks of health economics with a focus on vaccines highlights three critical aspects to consider when comparing vaccination with drug treatment.

First, most value measurements of vaccines used today in their economic assessments have been approached as if conducted for a drug evaluation programme. This implies a focus on the quality of the survival gain expressed as QALYs and/or DALYs. Vaccines have much more to offer than survival benefits amongst those at risk, as they also benefit populations not at risk or unvaccinated. The issue is to identify the best way to integrate the additional values into the economic assessment of vaccines. We will discuss that problem and ways in which it might be tackled in the subsequent papers.

Second, vaccines are a preventative intervention that has its main impact at the population and societal level. We also know that healthcare operates in a market that is not optimal as in an open market. The price paid for medical goods does not reflect the value as perceived by the consumer. Value needs to be expressed differently in these non-market conditions. The first economic evaluations of public projects as non-market situations, such as transport facilities and water canalisation, were conducted using the method of CBA with benefits assessed in monetary terms. However, categorising healthcare as a public-sponsored programme has a problem in that when treatment interventions were evaluated, the benefit was measured at the level of the individual and not the population. Furthermore, decision-makers, including the medical corps, had difficulties with assessing the health benefits of an individual in money terms. It is possible that CBA may be a more appropriate approach than ICUA for vaccines, as it could include the wider benefits of vaccines beyond the individual.

Third, many differences exist between treatment and vaccination. Assessing the economic value correctly for each will require reasoning differently. The starting points are very different, as vaccines need substantial initial investment. The budget is the major driver in their introduction, and the cost offsets potentially available from vaccines in the developed world can substantially offset the initial investment by savings in hospitalisations, medical visits, diagnostics and medications. In addition, achieving high coverage in the target population requires good preparation and active outreach. Accessibility must therefore be high with no hurdles to complicate the process of receiving the vaccine. In addition, there is an element of social duty in being vaccinated, because high vaccine coverage reduces the spread of infection, reduces the misuse and resistance of anti-infective drugs, and this responsibility aspect should be reinforced.

These three elements – value, population and the many differences – should convince us to explore other approaches for economic assessment that are better able to capture the value of vaccines for the payer, consumer, producer and wider society.

However, if we are forced to work with the evaluation instruments that are now available, maybe one element should receive closer attention for reassessment when considering vaccines, and that is the threshold selection in the ICUA. One may remember that the threshold could be defined under a fixed budget based on the last intervention ranked of the incremental cost-effectiveness results that also fulfils the budget constraint [69]. The threshold may change if a new intervention has a better outcome than the last one approved. If a full view on all the interventions to be evaluated is not available, alternatives for defining the threshold have been proposed such as WTP, comparison with past decisions, using the Gross Domestic Product per capita, opportunity cost approach, or weighting the ICUA value in an evaluation process including other critical elements such as budget impact, or overall societal outcome of the intervention, among others. It should be clear that selections made so far were again based mainly on a patient searching for QALY gain through a therapeutic intervention. Vaccines should be considered in a broader context with a threshold selected that fulfils the demands of society rather than the individual patient benefit. Societal demand could be evaluated through techniques developed for defining the WTP in environmental questions where health and quality health gain were assessed. Alternatively, the conventional ICUA calculation could be weighted in an evaluation process where other value elements such as overall cost, budget impact, equity, access, population benefit, and/or societal benefit are also considered in the equation.

## Conclusion

The economic evaluation of a new prophylactic vaccine may differ from that of a new therapeutic drug. Current economic evaluation of vaccines uses a conventional approach that has traditionally been applied to treatments. Here we question whether this is appropriate, and whether current tools capture the full value benefit of vaccination. At least four broad aspects of difference – population rather than individual benefit, societal evaluation rather than healthcare only, budget focus as well as cost–effectiveness analysis, and time changing objectives for similar intervention – indicate that additional evaluation frameworks should be sought to improve the evaluation of vaccines. It is up to us, health economists, to develop a framework that can answer those additional needs from different stakeholders.

## Supplementary Material

Supplemental_files.zip

## Highlights

- Today, economic assessment of vaccines uses the same evaluation method as for therapeutic interventions, namely the incremental cost–effectiveness analysis. This approach is maybe not fully appropriate because vaccines target prevention and not treatment or cure.
- We define prevention and vaccination, and highlight the many differences between vaccine and treatment related to the product, the population to target, and the financing processes.
- Within the preventative area, vaccination belongs to the category of active, social prevention: one has to act to get vaccinated and the vaccine creates benefit or value not only for the vaccinees but also for the others (indirect benefit or externality). In health economics, value is measured separately from cost. Value is first defined and subsequently the methods to measure it. However, the initial financial investment for vaccines is often the key hurdle for starting a new vaccination programme, and less the value for money obtained through the incremental cost–effectiveness analysis.
- The paper helps understanding why the economic assessment should be different for vaccines compared with treatment. This has implications for decision-makers who have to focus their attention on budget assignment, volume to be used, and total value generation of the new vaccine compared with the existing situation.

## supplementary material

The supplementary material for this article can be accessed here.
